# Intracranial Hybrid Neurofibroma/Schwannoma Arising From the Olfactory Groove: A Report of an Extremely Rare Case and Review of the Literature

**DOI:** 10.7759/cureus.80941

**Published:** 2025-03-21

**Authors:** Tomoya Goto, Yuji Kibe, Takuma Oishi, Shoichi Deguchi, Kazuya Motomura, Koichi Mitsuya, Masakuni Serizawa, Takeshi Nagashima, Keiichi Ohshima, Takashi Sugino, Kenichi Urakami, Yasuto Akiyama, Ken Yamaguchi

**Affiliations:** 1 Division of Neurosurgery, Shizuoka Cancer Center Hospital, Nagaizumi, JPN; 2 Division of Pathology, Shizuoka Cancer Center Hospital, Nagaizumi, JPN; 3 Department of Neurosurgery, Nagoya University Graduate School of Medicine, Nagoya, JPN; 4 Division of Drug Discovery and Development, Shizuoka Cancer Center Research Institute, Nagaizumi, JPN; 5 Division of Cancer Diagnostics Research, Shizuoka Cancer Center Research Institute, Nagaizumi, JPN; 6 Division of Oncology, SRL Inc., Tokyo, JPN; 7 Division of Medical Genetics, Shizuoka Cancer Center Research Institute, Nagaizumi, JPN; 8 Division of Immunotherapy, Shizuoka Cancer Center Research Institute, Nagaizumi, JPN; 9 Office of the President Emeritus, Shizuoka Cancer Center, Nagaizumi, JPN

**Keywords:** hybrid nerve sheath tumor, intracranial tumor, neurofibroma, olfactory groove tumor, schwannoma

## Abstract

Hybrid nerve sheath tumors (HNSTs) are rare peripheral nerve sheath tumors that combine the features of schwannomas, neurofibromas, and perineuriomas. Intracranial HNSTs are extremely rare. Herein, we report the first case of an intracranial hybrid neurofibroma/schwannoma arising from the olfactory groove.

A 62-year-old woman presented with right-sided hemiparesis and a gait disturbance. Magnetic resonance imaging (MRI) revealed a 45×50 mm extra-axial mass in the left anterior cranial fossa, suggestive of a meningioma or schwannoma. The patient underwent bifrontal craniotomy with complete tumor removal. Histopathological and immunohistochemical studies confirmed that the tumor consisted of two components, a neurofibroma and a schwannoma, resulting in the diagnosis of a hybrid neurofibroma/schwannoma. Whole-genome sequencing of tumor DNA revealed somatic mutations in *KMT2A* and trisomies of chromosomes 5 and 14q. No alterations were observed in *NF1*, *NF2*, or chromosome 22, and no germline mutations were identified. These results are not consistent with those of previously reported peripheral HNSTs, suggesting that the molecular biology of intracranial HNSTs may be different from that of peripheral HNSTs. The patient was discharged with no neurological deficits, and no recurrent findings were observed on follow-up MRI after one year.

To our knowledge, this is the first reported case of an olfactory groove HNST. We highlight the importance of further studies to elucidate its pathogenesis and genetic underpinnings.

## Introduction

The olfactory groove, located within the ethmoid bone of the anterior cranial fossa, is a relatively shallow concavity that contains the olfactory bulb connected to the olfactory nerve. While neuroblastomas and meningiomas are common tumors in this region, schwannomas are known to be rare due to the absence of Schwann cells in the olfactory nerves. To date, approximately 50 cases of olfactory schwannomas have been reported in the literature [1].

Nerve sheath tumors commonly occur in peripheral nerves and are mainly classified into schwannomas, neurofibromas, and perineuriomas. Schwannomas and neurofibromas originate from Schwann cells, each accounting for approximately 5% of benign soft-tissue neoplasms. Perineuriomas originate from perineurial cells, accounting for approximately 1% [2,3]. These tumors mostly develop sporadically, but it is known that neurofibromas and schwannomas are associated with neurofibromatosis type 1 (*NF1*) and neurofibromatosis 2 (*NF2*), respectively. These tumors are generally benign and do not usually recur if treated by gross total resection [3,4].

Hybrid nerve sheath tumors (HNSTs) were recently established as a distinct entity in the 4th edition of the World Health Organization (WHO) Classification of Tumors of Central Nervous System [3]. These tumors combine the histological features of conventional nerve sheath tumors, including schwannomas, neurofibromas, and perineuriomas. The most frequent combinations are schwannoma/perineurioma, neurofibroma/schwannoma, while the rarest is neurofibroma/perineurioma [4]. HNSTs commonly arise on the skin; therefore, cases involving the central nervous system are extremely rare [5]. To the best of our knowledge, only two cases of intracranial HNSTs, specifically hybrid schwannomas and perineuriomas, have been reported [6,7]. 

We encountered an exceptionally rare case of an intracranial HNST at the olfactory groove. Histopathological analysis revealed two distinct morphological and immunophenotypic components, leading to the diagnosis of a hybrid neurofibroma/schwannoma. Whole-genome sequencing (WGS) of tumor DNA revealed somatic mutations in *KMT2A *and trisomies of chromosomes 5 and 14q. No alterations were observed in *NF1*, *NF2*, or chromosome 22, and no germline mutations were identified. To our knowledge, this is the first report of a hybrid neurofibroma/schwannoma arising from the olfactory groove and the first report employing next-generation sequencing (NGS) for intracranial or nasal HNSTs.

## Case presentation

Clinical history and presentation

A 62-year-old woman presented with right-sided hemiparesis and gait disturbance. The patient exhibited no visual or olfactory dysfunction, aphasia, or other cognitive impairment. Neurological examination revealed no cranial nerve dysfunction and no motor deficits, except for mild right-sided hemiparesis, predominantly affecting the lower extremity. No cutaneous lesions were observed, and there was no history of cataracts or a familial history of neurofibromatosis or schwannomatosis.

Radiographic findings

**Figure 1:**
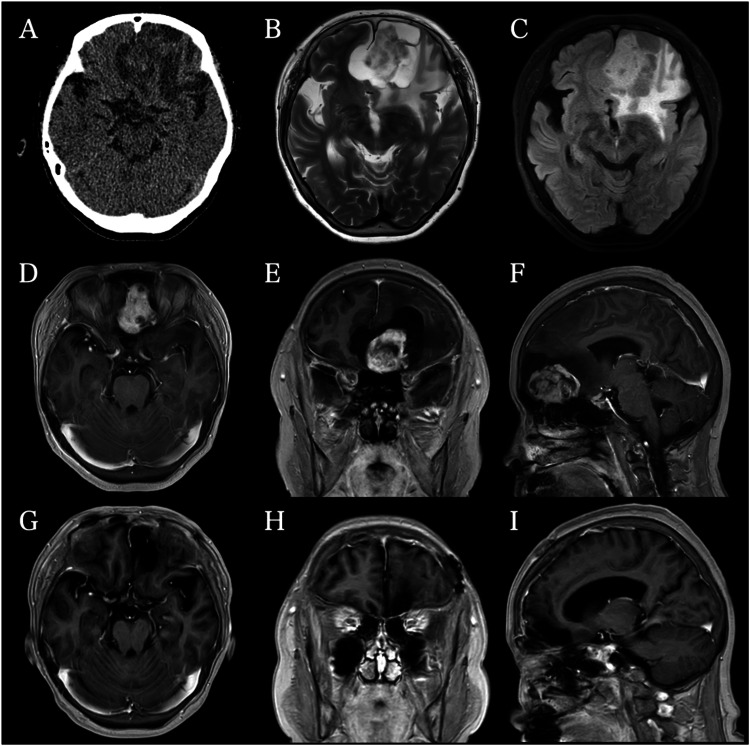
Radiographic Findings of an Olfactory Hybrid Neurofibroma/Schwannoma Preoperative imaging. (A) Axial CT demonstrating the mass lesion in the left anterior cranial fossa. (B, C) Axial T2-weighted and FLAIR MRI images depicting the large extra-axial tumor with peritumoral cysts and perifocal edema.  (D: axial, E: coronal, F: sagittal) On gadolinium contrast, the lesion demonstrated lobulated and heterogeneous enhancement, which seemed to be attached to the left cribriform plate. (G: axial, H: coronal, I: sagittal) Follow-up MRI one year after surgery demonstrated no recurrence.

Head computed tomography (CT) and magnetic resonance imaging (MRI) revealed a mass measuring approximately 45×50 mm on T2-weighted axial images, primarily located in the left anterior cranial fossa (Figures 1A-1C). The lesion appeared attached to the left cribriform plate and was identified as an extra-axial tumor with peritumoral cysts and a midline shift accompanied by perifocal brain edema. The lesion was not completely attached to the right olfactory groove. The mass was isointense on T1-weighted images (T1WI) and diffusion-weighted imaging (DWI) and hyperintense on T2-weighted images. T1-weighted gadolinium images showed heterogeneous enhancement (Figures 1D-1F). No invasion of the frontal base or nasal cavity was observed.

Surgical procedure

**Figure 2:**
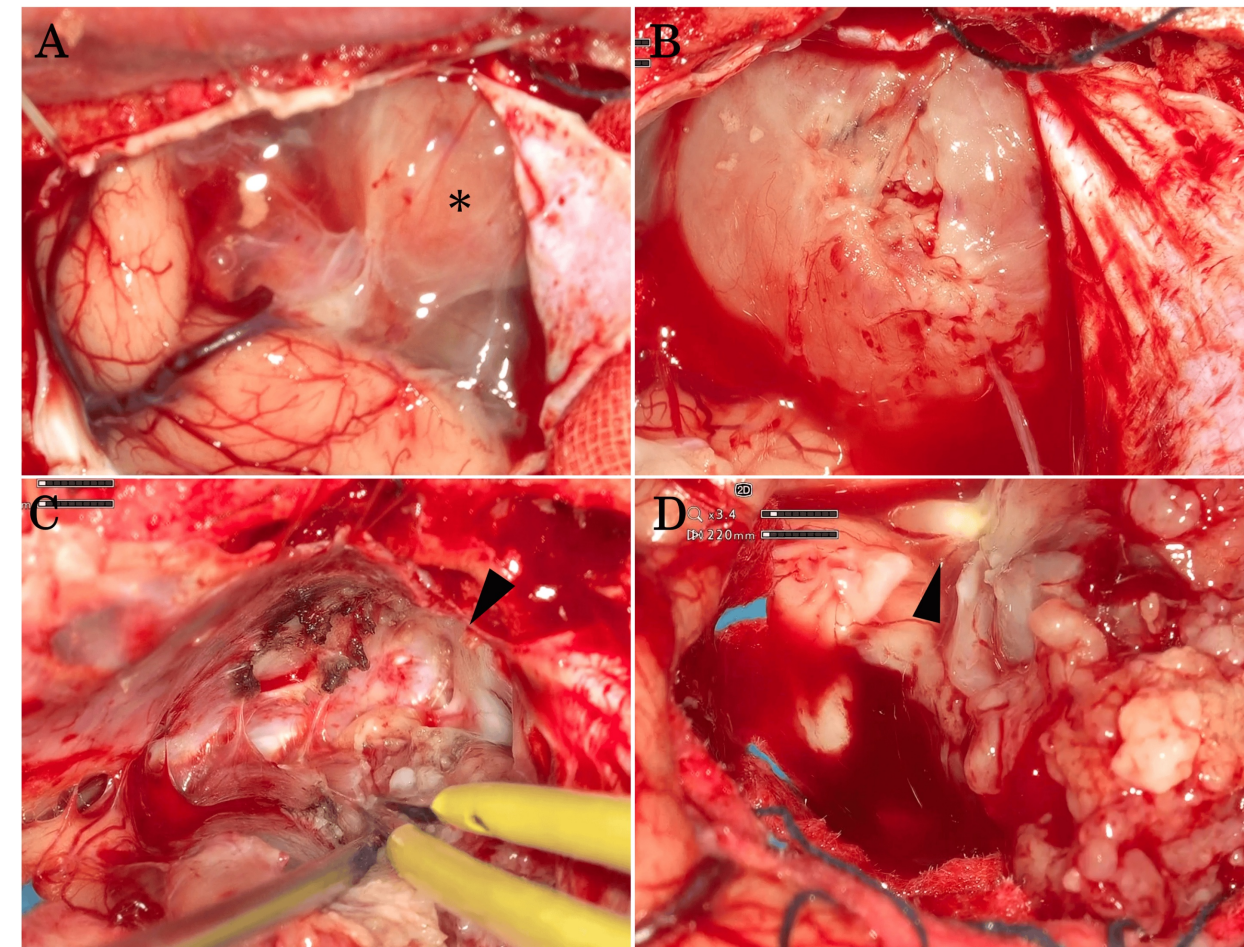
Intraoperative Images of an Olfactory Hybrid Neurofibroma/Schwannoma Intraoperative view of the tumor. (A) The border between the tumor (asterisk) with cysts and the left frontal lobe. (B) The surface and capsule of the tumor. (C) Attachment of the tumor to the left olfactory groove (arrowhead). (D) Encasement of the left olfactory tract (arrowhead). In all images, the upper, lower, left, and right sides indicate the caudal, cranial, left, and right sides of the patient, respectively.

As the tumor was located near the midline of the frontal base and attached to the bilateral frontal lobes, a bifrontal craniotomy with neuronavigation guidance was performed for tumor removal. The cyst wall of the tumor was observed and decompressed with drainage of the intracystic fluid (Figure 2A). Intraoperative findings revealed that the tumor had a well-defined capsule and was located in the intradural and extra-axial spaces (Figure 2B). The tumor firmly adhered to the left olfactory groove (Figure 2C), encasing the left olfactory tract (Figure 2D), suggesting its origin from the left olfactory nerve, while the right olfactory nerve was not attached to the tumor. There was no hypervascular component within the tumor, and bleeding was minimal. The tumor was dissected from the surrounding frontal lobe and completely removed with tumor capsule and the dura matter at the left olfactory groove, while preserving the arachnoid of the frontal lobes and the right olfactory nerve.

Postoperative course

Postoperatively, the patient was transferred to the intensive care unit in a stable neurological condition. MRI conducted on the following day confirmed complete tumor resection with no complications. Gradual improvement in the right-sided hemiparesis was observed postoperatively. However, cerebrospinal fluid (CSF) leakage from the nose occurred on postoperative day two. Management included continuous lumbar drainage, prophylactic ceftriaxone (CTRX), and complete bed rest. After cessation of the CSF leak was confirmed, the patient was discharged on postoperative day 11 with no neurological deficits. A follow-up MRI at one year showed no recurrence (Figures 1G-1I).

Pathological findings

**Figure 3:**
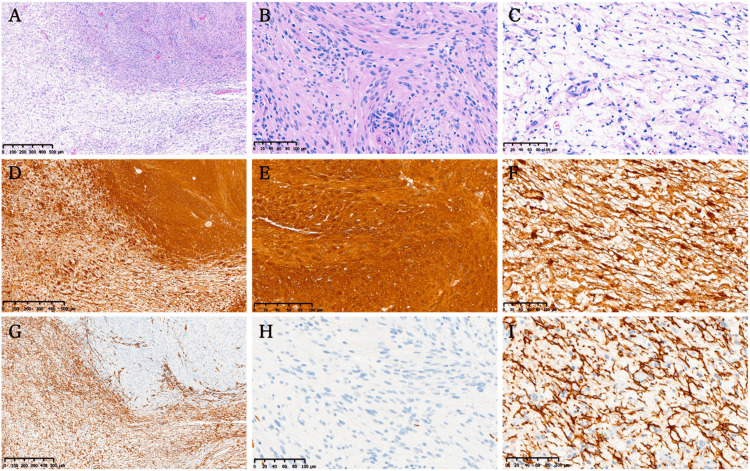
Pathological Findings of an Olfactory Hybrid Neurofibroma/Schwannoma Histopathological examination of the excised specimen (A): shows two distinctive areas with schwannomatous component (B) and neurofibromatous component (C). Both components are positive for S-100 (D: low power magnification, E: schwannomatous component, F: neurofibromatous component), however CD34 is positive only in the neurofibromatous component (G: low power magnification, H: schwannomatous component, I: neurofibromatous component). Scale bars represent 500µm (A, D, G) and 100µm (B, C, E, F, H, I).

Macroscopically, the tumor measured approximately 35 mm, as it was resected in a piecemeal fashion. The tumor was gray-white, slightly firm in texture, and contained cystic components. It was well-circumscribed by normal tissues. Microscopic examination revealed that the presence of a capsule and its relationship with normal tissues were unclear in the prepared specimens. The tumor was composed of two distinct morphological components (Figure 3A). One component was a hypercellular area with spindle cells, consistent with schwannomas (Figure 3B), while the other was a hypocellular area with spindle cells along with wavy to oval nuclei embedded in fibrillar and myxoid backgrounds, consistent with neurofibromas (Figure 3C). Schwannomatous components formed focal segments surrounded by fibromatous components, and the two components were intermingled without transitional parts. Each component constituted > 30% of the tumor, consistent with the diagnosis of an HNST. Minimal brisk mitosis and no necrosis were observed, although small calcifications were present. The stroma contained numerous blood vessels with hemorrhage and hemosiderosis. Immunohistochemical analysis revealed that the tumor cells in both components were diffusely positive for S-100 (Figures 3D-3F) and SOX-10. In contrast, CD34 expression was negative (Figures 3G, 3H) in the schwannomatous component, but positive in the neurofibromatous component (Figure 3I). The Ki-67 staining index was 6%. These findings confirmed the diagnosis of a hybrid neurofibroma/schwannoma.

Genetic findings

**Figure 4:**
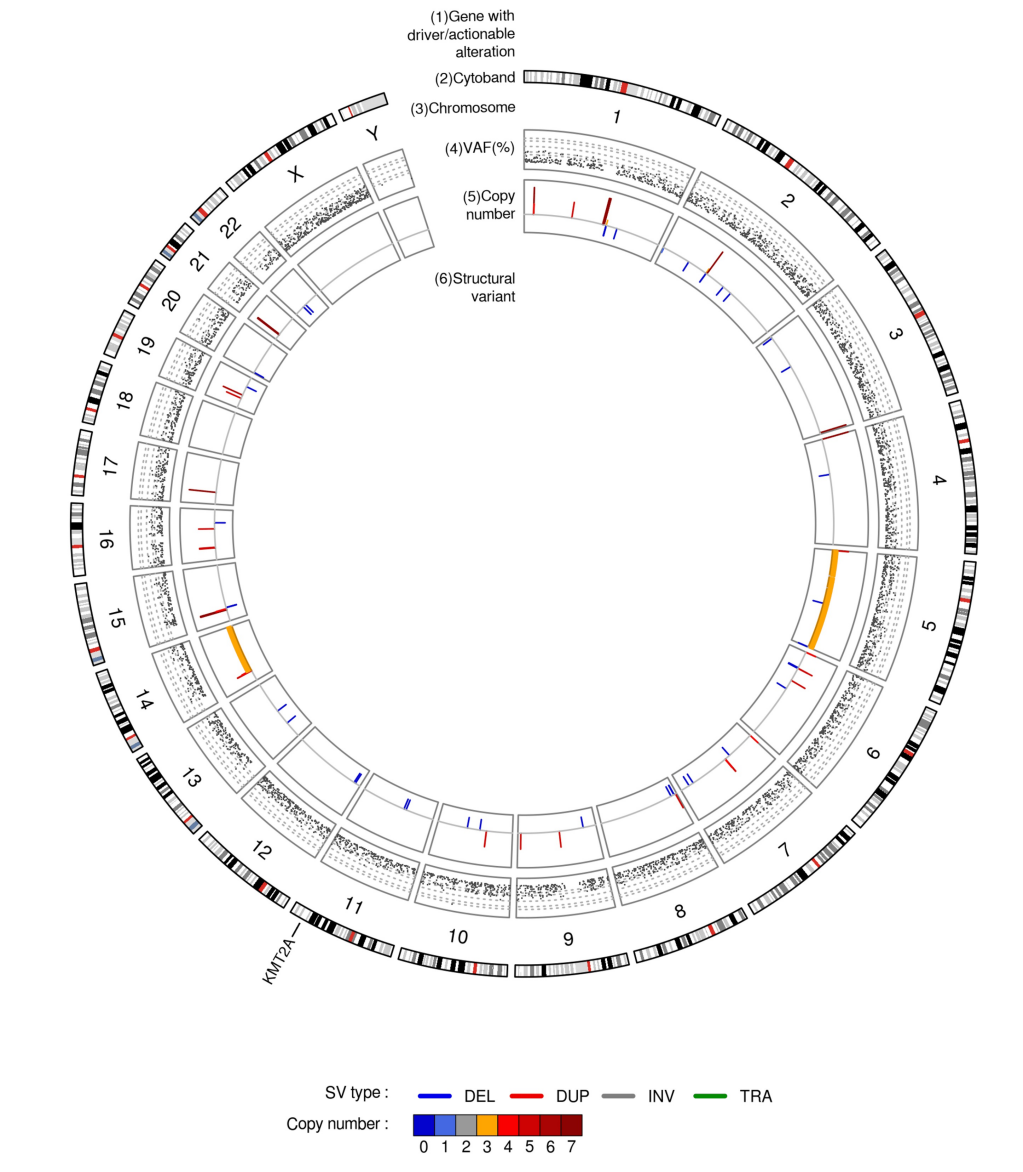
Genetic Findings of an Olfactory Hybrid Neurofibroma/Schwannoma Whole genome sequencing of an olfactory hybrid neurofibroma/schwannoma sample with structural aberrations. Circos plot shows the structural variations. The outer ring indicates the mutations, and the inner ring indicates the copy number variations (red: gain; blue: loss).

WGS was performed as a matched pair analysis, with a coverage of 137.23x for the tumor samples and 41.34x for the normal samples. WGS identified somatic mutations in *KMT2A* (c.4408C>T, p.Q1470*). No mutations were detected in *NF1* or *NF2*. Copy number analysis revealed trisomies of chromosomes 5 and 14q, with no abnormalities in chromosome 22. Germline mutations were not identified (Figure 4).

## Discussion

HNSTs are benign peripheral nerve sheath tumors characterized by the combined histological features of more than one conventional subtype, such as schwannoma, neurofibroma, and perineurioma. While HNSTs have been reported across a wide age range, they are most frequently observed in young adults and do not exhibit any sex predilection. However, their exact incidence remains unknown due to their rarity [5]. The most common subtype is hybrid schwannoma/perineurioma, followed by hybrid neurofibroma/schwannoma [4]. Feany et al. first described nine cases of hybrid neurofibroma/schwannoma in 1998, all of which occurred in the extremities and trunk [8]. While most reported cases of HNSTs are located in the digits or extremities, atypical sites such as the colon, orbit, nasal cavity, and spinal nerve have also been documented [9-13]. Hong et al. summarized six cases of orbital HNSTs, all of which were hybrid neurofibromas and schwannomas [10]. Intracranial HNSTs are extremely rare. To date, only two cases have been reported in the literature: a 24-year-old woman with a hybrid schwannoma/perineurioma in the internal auditory canal [6] and a 22-year-old woman with a trigeminal hybrid schwannoma/perineurioma [7]. Other reported cases include one nasal and two spinal HNSTs [11-13], none of which were analyzed using WGS data (Table 1). The patient in this case report is significantly older than the previously reported intracranial cases, likely because the tumors in the olfactory groove allowed it to remain asymptomatic until it had grown substantially. In these two intracranial cases, Schwann cells may have been involved in the origin of the tumor, although the origin of the HNSTs remains unknown. The olfactory nerve does not contain a Schwann cell layer; therefore, peripheral nerve sheath tumors, including schwannomas, cannot originate from the olfactory nerve. Therefore, while HNSTs are rare by nature, their occurrence in the olfactory groove represents an even more unusual phenomenon. In contrast, intracranial schwannomas are relatively common intracranial tumors, which account for 6-8% of all intracranial tumors [14]. These tumors predominantly arise from the eighth cranial nerve, with olfactory schwannomas being particularly rare. Hadjigeorgiou et al. summarized 53 cases of olfactory groove schwannomas [1]. The origin of olfactory groove schwannomas remains unclear; however, two hypotheses have emerged: the developmental theory and the non-developmental theory. Developmental theory explains the transformation of mesenchymal pial cells into ectodermal Schwann cells or the migration of neural crest cells within the central nervous system. The non-developmental theory postulates that these tumors can arise from normal Schwann cells in the perivascular plexus and meningeal branches of the anterior ethmoidal nerve, trigeminal nerve, or filia olfactoria [15-18]. These theories may also explain the origin of the olfactory groove in the HNST. Additionally, the hybrid feature of neurofibromas and schwannomas suggests that these two tumors are more closely related than previously understood [8].

**Table 1:** Reported Cases of Intracranial, Nasal, and Spinal Hybrid Nerve Sheath Tumors

Author, year	Age, sex	Tumor size (cm)	Tumor location	Characteristics of tumor on MRI	Pathologic components	Stigma of neurofibromatosis	Gene alterations
Las Heras et al. 2013 [6]	24, female	1.3	Intracranial (left internal auditory canal)	Solid and cystic	Schwannoma/perineurioma	None (there is a history of multiple sclerosis)	Not available
Goyal-Honavar et al. 2023 [7]	22, female	5.2	Intracranial (right cerebellopontine angle)	Solid	Schwannoma/perineurioma	None	Not available
Kuroda et al. 2010 [11]	58, male	Unknown	Nasal cavity	Unknown	Neurofibroma/schwannoma/perineurioma	None	Not available
Hayashi et al. 2013 [13]	63, male	1.5/2.2	Spinal nerve (nerve root, cauda equina)	Both solid	Both schwannoma/perineurioma	None	Not available
Inomata et al. 2022 [12]	56, male	Unknown	Spinal nerve (nerve root)	Solid	Neurofibroma/schwannoma	None	Not available
The present study	62, female	5.0	Intracranial (left olfactory groove)	Solid and cystic	Neurofibroma/schwannoma	None	KMT2A mutation, trisomies in 5 and 14q

Histopathologically and immunohistochemically, the tumor consisted of two distinct components: neurofibroma and schwannoma. In the neurofibromatous component, tumor cells were small, spindle-shaped with wavy to oval nuclei embedded in myxoid stroma with variable collagen deposition. A compact pattern (Antoni A) was identified in the schwannomatous components, characterized by increased cellularity and spindle nuclei. Both components were diffusely positive for S100 and SOX10, markers of mature Schwann cells. However, CD34 and EMA, which are markers for perineurial and stromal cells, respectively, are positive in the neurofibromatous area, but negative in the schwannomatous area [4]. Each component was intermingled and there were no transitional parts. This case showed relatively typical features of a hybrid neurofibroma/schwannoma and was pathologically diagnosed as a hybrid neurofibroma/schwannoma despite its extremely rare location.

Hybrid neurofibromas/schwannomas are associated with *NF1*, *NF2,* and schwannomatosis, whereas hybrid schwannomas/perineuriomas occur sporadically [19]. In contrast, our patient had no cutaneous lesions and did not meet any of the criteria for these tumor predisposition syndromes. Furthermore, WGS did not identify any germline mutations, indicating sporadic cases without neurofibromatosis or schwannomatosis.

Although the molecular genetic events remain unclear, some genetic findings in hybrid neurofibromas and schwannomas have been reported. *ERBB2 *mutation in hybrid neurofibromas and schwannomas has been proposed as a potential diagnostic and therapeutic target [20]. Additionally, Stahn et al. identified 22 monosomy in approximately half of hybrid neurofibromas/schwannomas and implicated the involvement of the *CTNNA3 *(α-T-catenin) gene in HNSTs [21]. However, these alterations were not observed in our patient. Our WGS analysis passed quality control with sufficient coverage depth; hence, the absence of previously reported alterations in our case was not due to analytical problems. A possible explanation is that intracranial HNSTs are biologically different from the peripheral HNSTs reported in the aforementioned literature. Molecular analyses using NGS were not performed for any of the reported intracranial HNSTs. We performed WGS of DNA from the tumor in this case for the first time and identified a nonsense variant of *KMT2A*. *KMT2A *plays a key role in regulating gene expression as a transcriptional coactivator, and its mutations have been reported primarily in blood malignancies [22] but not in schwannomas or neurofibromas. Our data also revealed trisomies of chromosomes 5 and 14q. Chromosome 5 contains several oncogenes, including *MDM2*, *TERT, *and *PDGFRB*. Gains of chromosome 5p have been reported in several cancers, such as cervical cancer and lung cancer [23,24]. Additionally, there is a report of chromosome 5p gains in malignant peripheral nerve sheath tumors (MPNSTs) [25]. However, the contribution of this chromosomal gain to the pathogenesis of MPNSTs and other nerve sheath tumors remains poorly understood. Chromosome 14q contains several oncogenes, including *HIF1A *and *AKT1*. While numerous studies have reported the loss of chromosome 14q in various cancers [26,27], there are few reports regarding 14q gains. Consequently, the pathophysiological significance of this alteration remains unclear. Furthermore, the tumor in our case also did not harbor alterations commonly reported in intracranial schwannomas, such as *ARID1A/B*, *DDR1*, or *SOX10* mutations [28], suggesting that it might arise from a different mechanism than intracranial schwannomas. Overall, there are few reports about genetic features of intracranial HNSTs. Thus, further accumulation of HNST cases and detailed genetic analysis are needed to elucidate the molecular biology of intracranial HNSTs.

Due to their rarity, preoperative prediction of intracranial HNSTs is nearly infeasible. However, the possibility of nerve sheath tumors, primarily schwannomas, should be considered when encountering a well-circumscribed extra-axial tumor at the olfactory groove. In pathological diagnosis, unlike schwannoma, neurofibroma contains fibroblasts in the background of neoplastic Schwann cells. Therefore, identifying the presence of collagen fibers or fibroblasts in the tumor component is possibly the first step in diagnosing hybrid neurofibroma/schwannoma. In addition to morphological differences, the expression pattern of immunohistochemical markers, such as CD34 or EMA, which are positive only for the neurofibromatous component, is useful for differential diagnosis of nerve sheath tumors including HNSTs. Although the long-term outcome of intracranial HNSTs remains unclear, surgical excision seems to be the first-line treatment, as peripheral HNSTs are usually benign and rarely recur after complete excision [3]. However, malignant transformation has been described in only a few cases, and postoperative follow-up is necessary [5]. In this case, total tumor removal was achieved and no recurrence was confirmed after one year.

## Conclusions

Intracranial HNSTs are extremely rare. To the best of our knowledge, this is the first reported case of an HNST arising from the olfactory groove. Given that the olfactory nerve lacks Schwann cells and other peripheral nerve sheath-forming cells, the exact origin of our case's tumor remains unclear. Genetic analysis suggested that the molecular biology of intracranial HNSTs may be different from that of peripheral HNSTs. Further accumulation of cases and detailed research are needed to better understand the pathogenesis of this rare condition.
